# *MYBL2* alternative splicing-related genetic variants reduce the risk of triple-negative breast cancer in the Chinese population

**DOI:** 10.3389/fgene.2023.1150976

**Published:** 2023-04-18

**Authors:** Xinyu Chen, Jin Feng, Yuan Zhang, Jiarui Liu, Lijia Zhang, Pu Zeng, Langbo Wen, Xin Wang, Yi Zhang

**Affiliations:** ^1^ Department of Hygiene Toxicology, School of Public Health, Zunyi Medical University, Zunyi, China; ^2^ Department of Laboratory Medicine, Affiliated Hospital of Zunyi Medical University, Zunyi, China; ^3^ Department of Orthopaedic Surgery, Affiliated Hospital of Zunyi Medical University, Zunyi, China

**Keywords:** MYB proto-oncogene like 2, triple-negative breast cancer, alternative splicing, SNP, susceptibility

## Abstract

**Background:** Triple-negative breast cancer (TNBC) is the most malignant subtype of breast cancer, and studies have found an association between the Myb proto-oncogene like 2 (*MYBL2*) gene and TNBC development; however, the specific mechanisms underlying development remain unknown. Recent studies have reported the association of alternative splicing (AS) with cancer, providing new approaches to elucidate the carcinogenesis mechanism. This study aimed to identify *MYBL2* AS-related genetic variants that influence the risk of developing TNBC, providing new ideas for probing the mechanism of TNBC and novel biomarkers for TNBC prevention.

**Methods:** We conducted a case-control study of 217 patients with TNBC and 401 cancer-free controls. The CancerSplicingQTL database and HSF software were used to screen for *MYBL2* AS-related genetic variants. The association of sample genotypes with the risk of TNBC development and with clinicopathological features was analysed via unconditional logistic regression. Combining multiple platforms, the candidate sites were subjected to biological function analysis.

**Results:** Two AS-associated SNPs, rs285170 and rs405660, were identified using bioinformatics analysis. Logistic regression analysis showed that both rs285170 (OR = 0.541; 95% CI = 0.343–0.852; *p* = 0.008) and rs405660 (OR = 0.642; 95% CI = 0.469–0.879; *p* = 0.006) exhibited protective effects against TNBC under the additive model. Stratification analysis showed that these two SNPs had more significant protective effects in the Chinese population aged ≧50 years. Additionally, we found that rs405660 was associated with the risk of lymph node metastasis (OR = 0.396, 95% CI = 0.209–0.750, *p* = 0.005) in TNBC. Functional analysis revealed that both rs285170 and rs405660 are associated with splicing of exon 3 and that the exon 3-deleted spliceosome does not increase breast cancer risk.

**Conclusion:** We found for the first time that *MYBL2* AS-related genetic variants are associated with reduced TNBC susceptibility in the Chinese population, especially in women aged ≧50 years.

## 1 Introduction

In recent years, the incidence of breast cancer has been increasing annually; according to the American Cancer Society, the number of new cases of breast cancer worldwide was 2.26 million in 2020, which is the highest number of new cancer cases recorded to date (Sung et al., 2021). Breast cancer can be classified into four subtypes based on the expression of oestrogen receptor (ER), progesterone receptor (PR), human epidermal growth factor-2 (HER2), including luminal A, luminal B, and HER-2-overexpressing, and triple-negative breast cancer (TNBC; Burguin et al., 2021). Among them, triple negative breast cancer accounts for 15%–20%, which also results in poor prognosis due to its special molecular phenotype and insensitivity to endocrine or molecular targeted therapy. In general, the five-year survival rate for breast cancer can reach 90%; however, it is only 12% for metastatic TNBC (Burguin et al., 2021). Although treatment options for TNBC, such as specific receptor targeted therapies, immunotherapies, and antibody drug conjugate therapies, have been intensely investigated by researchers worldwide in recent years, some of which have achieved staged results, they are still far from being applied in clinical use (Yin et al., 2020; Bianchini et al., 2022). Therefore, the most effective treatment method involves prevention of TNBC development. Breast cancer prevention is now possible owing to the administration of preventive drugs and genetic screening; however, studies have shown that breast cancer preventive drugs recommended by international guidelines do not favourably reduce breast cancer mortality and only offer a certain preventive benefit in ER-positive breast cancer (Hong and Xu, 2022). Thus, in addition to continuing vigorous efforts to develop effective preventive drugs, studies on genetic predisposition to breast cancer are necessary. Breast cancer is typically a heterogeneous disease, and genetic variants play a large role in individual susceptibility. Thus, identification of genetic variants that influence breast cancer susceptibility may help reduce the risk of breast cancer.

Genetic susceptibility to breast cancer is increasingly investigated by post-genome-wide association studies, so that current research focuses on identifying functional genetic variants that affect breast tissue carcinogenesis and elucidating the underlying mechanisms via molecular biology experiments. Alternative splicing (AS) is a key molecular event during gene expression, and it involves generation of RNA exons from pre-mRNA transcription binding via selection of different splice sites, resulting in different mRNA splicing isoforms. It is an intricate mechanism of coordinated transcriptional regulation that enables a single gene to generate different mRNAs, thereby greatly increasing the diversity of the transcriptome and proteome. This phenomenon is present in almost all genes encoding proteins. AS occurs widely in human tissues; however, abnormal splicing events may lead to the development of various neurological diseases and cancers (Ren et al., 2021). At least 15,000 splice variants affect cancer development across 27 cancer types (Kahles et al., 2018). For example, the BRCA1--Δ11q variant affects breast carcinogenesis and drug resistance. Compared to the full-length BRCA1 spliceosome, BRCA1--Δ11q clearly promotes cancer cell growth as well as resistance to PARP inhibitors and cisplatin (Nielsen et al., 2016; Wang et al., 2016). In addition, there is evidence for the existence of specific spliceosomes in BC tissues that are not found in normal tissues (Dutertre et al., 2010). Aversa et al. (2016) also found 12 novel AS transcripts in BC tissues based on RNA-seq data. These abnormal ASs were closely related to the occurrence of breast cancer (Yang et al., 2019). Further studies on AS have high potential to disentangle the biological mechanisms of breast cancer.

The MYB proto-oncogene like 2 (*MYBL2*) gene, a member of the MYB family of transcription factors and one of the key genes regulating cell cycle progression, is expressed in almost all proliferating cells and regulates cell survival and differentiation, while also mediating aberrant cell progression in cancer (Ness, 2003). Reddel, (2000) discovered that cell immortalisation can be achieved by dysregulated proliferation and that *MYBL2* amplification is the earliest observed event during this process. In addition, bioinformatics data mining revealed that dysregulation of genes, as key drivers in breast cancer, controls the cell cycle, in which *MYBL2* plays an important role (Lauss et al., 2008). Distinct amplification of *MYBL2* is found in approximately 9%–13% of patients with breast cancer, a feature that is particularly evident in TNBC (Thorner et al., 2009). Multiple studies have found that single nucleotide polymorphisms (SNPs) create functional alterations in *MYBL2*, which in turn affect disease initiation and progression. For example, the rs285207 polymorphism disrupts binding of *MYBL2* to transcription factor IKZF1, affects transcription of *MYBL2*, and in turn reduces susceptibility to childhood acute lymphoblastic leukaemia (Guo et al., 2021). *MYBL2* is phosphorylated by cyclin A/Cdk2 in the S phase, which activates the protein. When *MYBL2* is expressed and activated in the late G1 and S phases of the cell cycle, it directly binds to the promoter and expresses trans-activated genes in the G2/M phases (Musa et al., 2017). Several recognised transcription factor binding sites are located in the *MYBL2* promoter region, and the rs619289, rs826943, and rs826944 polymorphisms located in the promoter region affect *MYBL2* expression and confer susceptibility to breast cancer (Shi et al., 2011). Analysis of microarray data also found that the *MYBL2* rs2070235 polymorphism increases the risk of basal like breast cancer, but the specific mechanism remains unknown (Thorner et al., 2009). Thus, the aberrant expression of *MYBL2* caused by SNPs likely affects the occurrence and development of breast cancer.

Although multiple SNPs associated with breast carcinogenesis have been found, the association of *MYBL2* AS-related SNPs with breast cancer susceptibility has not been explored to date. SNPs have been reported to represent a major cause of AS, and AS of certain genes has been found to influence breast cancer susceptibility (Montgomery et al., 2010). Schmid et al. (2002) found that SNPs cause four different splice variants in the mucin 1 (*MUC1*) gene, and *in-vitro* assays showed that variants B and C are associated with invasiveness in breast cancer cell lines. A causal relationship between breast cancer risk-associated splicing events and SNPs has been confirmed in minigene experiments (Caswell et al., 2015). Based on these previous results, SNPs likely affect breast cancer susceptibility by affecting gene AS, leading to differential expression of different spliceosomes among individuals. The search for functional SNPs that mediate AS of *MYBL2* may provide a basis to explain individual differences in breast cancer susceptibility.

In this study, we systematically summarised all potential AS-causing SNPs of *MYBL2* via the CancerSplicingQTL database (Tian et al., 2019) and Human Splicing Finder (HSF) online software (Desmet et al., 2009). Moreover, we used a case-control study design to discover functional SNPs of this gene associated with TNBC risk. Our study provides new research ideas and methods for systematically discovering functional genetic variations. Our results provide insights into the mechanisms involved in the development and progression of TNBC and shed new light on screening of and individualised prevention for individuals at high risk for TNBC.

## 2 Materials and methods

### 2.1 Selection of AS-associated SNPs

All potential splicing-causing SNPs of *MYBL2* associated with breast cancer risk were searched for and downloaded from CancerSplicingQTL. And the coding DNA positions of these variants were obtained in the VannoPortal database. Moreover, linkage disequilibrium (LD) analysis between SNPs was performed using the Haploview 4.2 software. The potential AS function of these SNPs was further predicted in the HSF online software, and the minor allele frequency (MAF) of these SNPs in the southern Chinese population was detected using the 1000 Genomes Project. SNPs with an MAF >0.1 were screened, and redundant SNPs were removed according to HSF score and LD analysis.

### 2.2 Study participants and data collection

This study recruited 217 women with TNBC and 401 cancer-free women (controls) at the Affiliated Hospital of Zunyi Medical University from 2018 to 2021. All cases were histopathologically and immunohistochemically confirmed for TNBC; the immunohistochemical results met ER (-), PR (-), and HER-2 (-), and the patients had no previous history of other malignancies. The control group comprised cancer-free women attending the physical examination department of this hospital during the same period according to the table of random digit, and they were matched with the case group based on age (±5 years). Briefly, 2 ml of peripheral blood was drawn for subsequent testing. General information and pathological data of the study participants were obtained by querying medical records and conducting interviews. Data entry was completed using the EpiData 3.1 software. All data were entered in duplicate, and then, inter-input comparisons were performed to ensure the accuracy of data entry. Participants who had never smoked or smoked less than one cigarette per day were defined as “never smoked” and all other cases were defined as “smoked.” Alcohol consumption twice or more per week for at least one year was defined as “drinking” and other cases were considered as “no drinking.” The ethics committee of the Affiliated Hospital of Zunyi Medical University approved the study (No. KLLY-2021-200). All participants provided written informed consent.

### 2.3 DNA extraction and genotyping

The collected peripheral blood samples were used to extract genomic DNA using the BloodZol Kit (TransGen Biotech, Beijing, China). The concentration of the DNA solution was diluted to 50 ng/µL and stored at −20°C. Sample DNA was genotyped using the CFX96 real-time PCR detection system (Bio-Rad, Hercules, CA, United States) and TaqMan probes (SANGON, Shanghai, China). Duplicate experiments were performed using 5% of samples randomly selected from cases and controls; the results of the two experiments were compared for consistency.

### 2.4 Analysis of biological function

We used the CancerSplicingQTL database to analyse candidate sites, the exon in which they were predicted to act, the splicing pattern, and the splice site, to determine what spliceosome the candidate site might affect. The expression differences in *MYBL2* splice isoforms among different tissues were analysed using the TSVdb platform (http://tsvdb.com/index.html) to determine whether the splice isoforms are associated with breast cancer risk.

### 2.5 Statistical analysis

Calculation of power was performed using the following formula:

$$\begin{array}{l} n=\left[Z_{1-\frac{a}{2}}\sqrt{\left(1+1/r\right)\bar{p}\left(1-\bar{p}\right)}+Z_{\beta }\sqrt{p_{1}\left(1-p_{1}\right)/r+p_{0}\left(1-p_{0}\right)}\right]/{\left(p_{1}-p_{0}\right)}^{2}\\ p_{1}=\left(OR\times p_{0}\right)/\left(1-p_{0}+OR\times p_{0}\right)\\ \bar{p}=\left(p_{1}+rp_{0}\right)/\left(1+r\right) \end{array}$$

p_0_: Exposure rates of risk factor in controls; p_1_: Exposure rates of risk factors in the cases; r: Ratio of control sample size to case sample size

It was calculated that 200 cases and 400 controls would provide 91% test power when OR = 2, α = 0.05, and MAF = 0.1. Chi-square tests and two independent-sample t-tests were used to compare whether demographic statistical characteristics were significantly different between cases and controls. The chi-square goodness-of-fit test was used to test whether the genotype distribution in controls was consistent with the Hardy–Weinberg equilibrium, and a logistic regression model was used to calculate the odds ratio (OR) and 95% confidence interval (CI). Values of *p* < 0.05 were considered statistically different. All data were analysed using the SPSS 26.0 software (IBM, Armonk, NY, United States).

## 3 Results

### 3.1 Screening of genetic variants associated with MYBL2 alternative splicing

**FIGURE 1:**
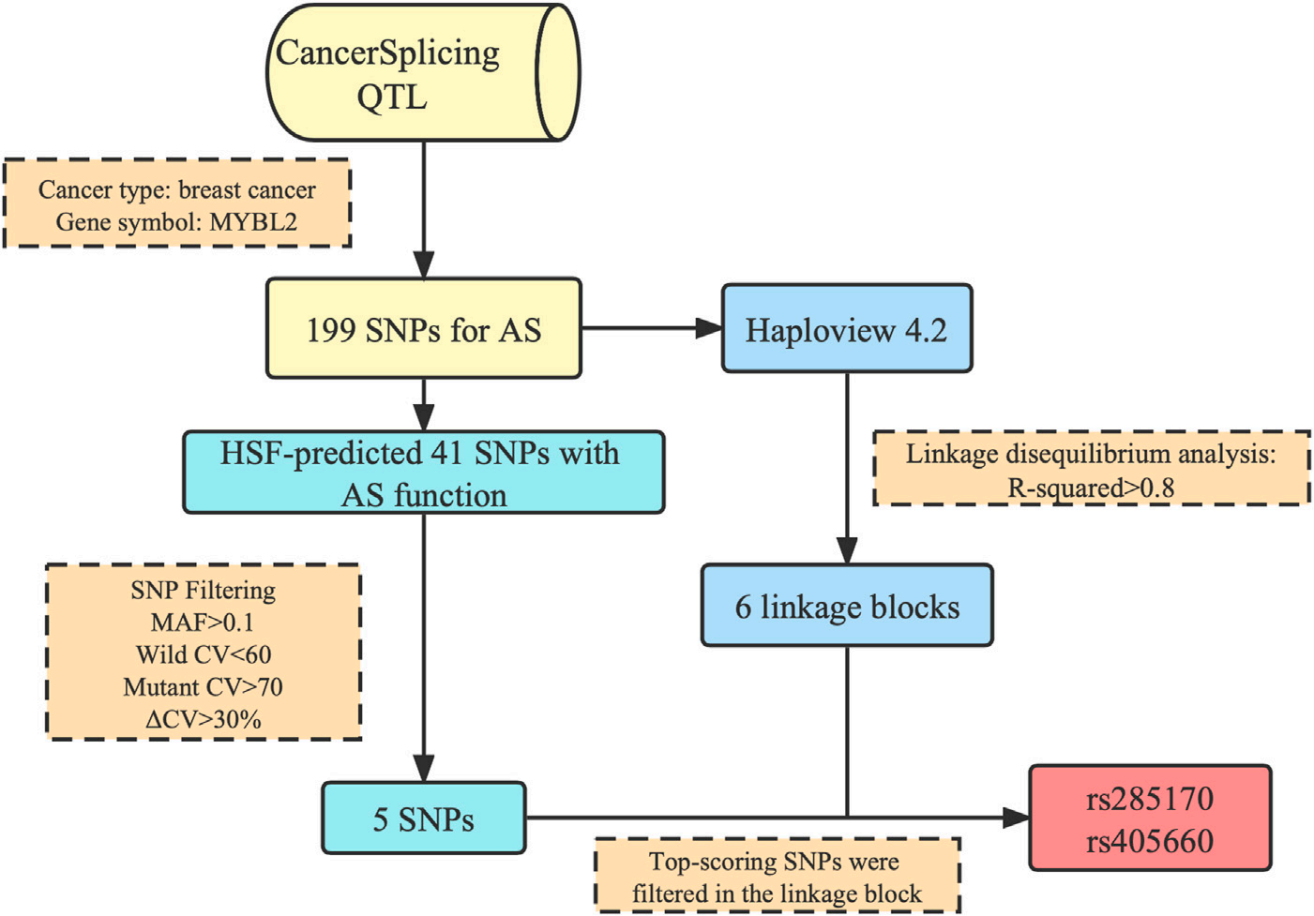
Flowchart of *MYBL2* alternative splicing-related SNP screening. 199 *MYBL2* AS-related genetic variants were acquired at CancerSplicingQTL. Five SNPs with potential functions in alternative splicing were further screened out in HSF. Six linkage blocks were simultaneously obtained by Haploview. Two SNPs, rs285170 and rs405660, were finally included in the follow-up study.

Through bioinformatics analysis (Figure 1), we obtained a total of two SNPs (rs285170 and rs405660) potentially associated with *MYBL2* AS in BC, and the specific screening pipeline was as described below.

#### 3.1.1 Initial prediction of MYBL2 AS‐related genetic variants

Analysing the association of splicing events with SNPs occurring in tumour tissues of TCGA breast cancer patients, we first selected SNPs within the breast cancer category among the 253767 SNPs potentially affecting AS in breast cancer. We then delimited the *MYBL2* gene, which finally resulted in 199 breast cancer associated SNPs potentially affecting AS in *MYBL2* in CancerSplicingQTL (Supplementary Table S1).

#### 3.1.2 LD analysis

**FIGURE 2:**
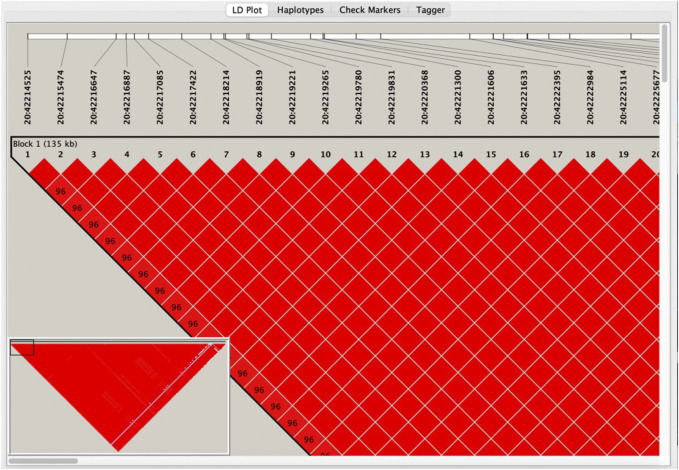
Visualisation of linkage disequilibrium analysis by Haploview.

LD analysis was performed using Haploview to guarantee that the last SNP included in the study was genetically independent. After downloading the genotyping data of all SNPs on *MYBL2* in Southern China from the Ensembl database, the data of the 199 SNPs from the first step were filtered out, collated into ped and info files (file format required for Haploview LD analysis), and input into Haploview. The r^2^ values among loci (Supplementary Table S2) and six linkage blocks (Figure 2) were obtained.

#### 3.1.3 AS functional prediction and determination of candidate loci

To further verify whether the above 199 SNPs have AS function, we employed HSF for AS function prediction. We used the Vannoportal database to query transcript IDs and HGV numbers corresponding to the 199 SNPs obtained in the first step and then predicted the data using HSF, which indicated that a total of 41 SNPs were likely to affect *MYBL2* AS (Supplementary Table S3). To compare the strength of splicing activity at each locus, only SNP loci with HSF scores were included in this study. Of the 41 potentially AS functional sites, ten SNPs were predicted by HSF to affect splice donor or splice acceptor generation. Based on the scores given by the HSF predictions, we further filtered SNPs with wild-type CV values <60, mutant CV values >70, ΔCV >30%, and AS MAF >0.1, finally yielding a total of five SNPs. Combined with the results of the LD analysis, only two of the six linkage blocks contained SNPs that satisfied the filtering criteria, and two of the best scoring SNPs, rs285170 and rs405660, were finally screened out in each of these two linkage blocks, respectively (Table 1).

**TABLE 1:** Outcome of HSF prediction.

Position (hg38)	Gene symbol	SNP ID	Allele	Functional prediction^a^	HSF score^b^	MAF
Chr20:43680075	MYBL2	rs405660	G/T	Acceptor site	57.82 > 85.69 => 48.2%	T = 0.1952
Chr20:43707040	MYBL2	rs285170	T/C	Donor site	52.62 > 79.76 => 51.58%	C = 0.1048

^a^ Functional prediction by HSF.

^b^ HSF, Score: Wild CV > Mutant CV => ∆CV.

### 3.2 General demographic characteristics of the study participants

A total of 217 TNBC cases and 401 cancer-free controls were included in our case-control study, with a mean age of 50.41 years (±10.93 years) in the case group and 50.28 years (±11.57 years) in the control group. There was no significant difference in age (*p* = 0.889) between cases and controls. After stratification based on age, there was still no significant difference in age between cases and controls. There were also no significant differences in smoking and alcohol consumption between cases and controls. The TNM staging characteristics are described in Table 2.

**TABLE 2:** Study participants basic demographic data.

Variable	Case (%) (N = 217)	Control (%) (N = 401)	*P*
Age (Mean ± Std)	50.41 ± 10.93	50.28 ± 11.57	0.889^a^
<50	106 (48.85)	196 (48.88)	
Age (Mean ± Std)	41.83 ± 6.78	41.17 ± 7.38	0.445^a^
≧50	111 (51.15)	205 (51.12)	
Age (Mean ± Std)	58.60 ± 7.23	58.99 ± 7.39	0.659^a^
Cigarette smoking			
No	207 (95.39)	383 (95.51)	0.946^b^
Yes	10 (4.61)	18 (4.49)	
Alcohol consumption			
No	216 (99.54)	397 (99.00)	0.477^b^
Yes	1 (0.46)	4 (1.00)	
TNM	195 (89.86)		
Tumor size			
≤5 cm	167 (76.96)		
>5 cm	28 (12.90)		
Node			
N0	87 (44.62)		
>N0	108 (55.38)		
Metastasis			
M0	181 (92.82)		
M1	14 (7.18)		

^a^ The two groups were compared with independent *t*-test.

^b^ Chi-square test.

### 3.3 Association of MYBL2 AS-related genetic variants with TNBC susceptibility

Typing experiments were performed in which 5% of the samples were randomly selected for repeated experiments with an anastomosis rate of 100%. The distribution of rs285170 typing results of cases and controls is shown in Table 3. The genotype distributions of both SNPs in the control group were in accordance with HWE.

**TABLE 3:** Association between AS-related genetic variants and TNBC.

	Case	Control	HWE	OR (95%CI); *P* ^b^
	No(%)	No(%)	χ^2^;*P* ^a^	
rs285170	217 (100.00)	401 (100.00)	0.120; 0.729	
TT	191 (88.02)	318 (79.30)		1.000
TC	25 (11.52)	79 (19.70)		0.526 (0.323-0.855);**0.009**
CC	1 (0.46)	4 (1.00)		0.416 (0.046-3.746); 0.434
Dominant				0.520 (0.323-0.839);**0.007**
Addictive				0.541 (0.343-0.852);**0.008**
rs405660	216 (99.54)	398 (99.25)	2.044; 0.153	
GG	154 (71.30)	250 (62.81)		1
GT	60 (27.78)	125 (31.41)		0.781 (0.541-1.129); 0.188
TT	2 (0.92)	23 (5.78)		0.142 (0.033-0.609);**0.009**
Dominant				0.682 (0.476-0.976);**0.036**
Addictive				0.642 (0.469-0.879);**0.006**

^a^ The chi-square test of goodness of fit was performed to check for Hardy–Weinberg equilibrium of the control group.

^b^ ORs, and 95% CIs, were calculated by unconditional logistic regression analysis, adjusted for age, Cigarette smoking, and Alcohol consumption.

The bold values indicate that the results are statistically significant.

After logistic regression model analysis, the rs285170\C allele was found to be significantly associated with a decreased risk of TNBC. In addition, the dominant model (OR = 0.520; 95% CI = 0.323–0.839; *p* = 0.007) and additive model (OR = 0.541; 95% CI = 0.343–0.852; *p* = 0.008) both revealed that the C allele was a protective factor for TNBC; rs285170 (T > C) was a protective mutation for TNBC. Furthermore, we found that the rs405660GG genotype was significantly associated with a decreased risk of TNBC (OR = 0.142; 95% CI = 0.033–0.609; *p* = 0.009). The G allele exhibited protective effects against TNBC in the population under the dominant model (OR = 0.682; 95% CI = 0.476–0.976; *p* = 0.036) and additive model (OR = 0.642; 95% CI = 0.469–0.879; *p* = 0.006) (Table 3).

### 3.4 Stratified analysis of AS-related genetic variants and TNBC susceptibility

An age-stratified analysis was performed based on whether participants were aged ≧50 years. Notably, rs285170 was not significantly associated with TNBC susceptibility in participants aged <50 years; however, it showed a greater and significant difference in participants aged ≧50 years as compared to the whole population. In participants aged ≧50 years, the TC genotype was significantly associated with a decreased risk of TNBC (OR = 0.216; 95% CI = 0.088–0.529; *p* = 0.001), while the dominant (OR = 0.205; 95% CI = 0.084–0.503; *p* = 0.001) and additive (OR = 0.211; 95% CI = 0.087–0.510; *p* = 0.001) models similarly showed significant differences.

Similar to rs285170, we performed an age-based stratification analysis for rs405660. The G allele was not clearly associated with risk of TNBC in participants aged <50 years. However, in participants aged ≧50 years, the G allele showed a significant protective effect. The OR was significant under both dominant (OR = 0.485; 95% CI = 0.284–0.827; *p* = 0.008) and additive (OR = 0.461; 95% CI = 0.285–0.746; *p* = 0.002) models (Table 4).

**TABLE 4:** Stratification analysis of AS-related genetic variants and TNBC.

	Case (217)	Control (401)	HWE	OR (95%CI) (%); *P* ^b^
	No(%)	No(%)	χ^2^; *P* ^a^	
**rs285170**	217 (100.00)	401 (100.00)		
**<50**	106 (48.85)	196 (48.88)	0.027; 0.869	
TT	86 (81.13)	156 (79.59)		1.000
TC	19 (17.93)	38 (19.39)		0.915 (0.496-1.686); 0.776
CC	1 (0.94)	2 (1.02)		0.860 (0.077-9.657); 0.903
Dominant				0.912 (0.501-1.660); 0.763
Addictive				0.917 (0.526-1.599); 0.761
**≧50**	111 (51.15)	205 (51.12)	0.104; 0.747	
TT	105 (94.59)	162 (79.02)		1.000
TC	6 (5.41)	41 (20.00)		0.216 (0.088-0.529);**0.001**
CC	0 (0.00)	2 (0.98)		--
Dominant				0.205 (0.084-0.503);**0.001**
Addictive				0.211 (0.087-0.510);**0.001**
**rs405660**	216 (99.54)	398 (99.25)		
**<50**	105 (48.61)	195 (48.99)	0.801; 0.371	
GG	68 (64.76)	122 (62.56)		1
GT	35 (33.33)	63 (32.31)		0.977 (0.586-1.629); 0.928
TT	2 (1.91)	10 (5.13)		0.348 (0.074-1.636); 0.181
Dominant				0.890 (0.542-1.464); 0.647
Addictive				0.826 (0.538-1.269); 0.383
**≧50**	111 (51.39)	203 (51.01)	1.273; 0.259	
GG	86 (77.48)	128 (63.06)		1
GT	25 (22.52)	62 (30.54)		0.586 (0.341-1.009); 0.054
TT	0 (0.00)	13 (6.40)		--
Dominant				0.485 (0.284-0.827);**0.008**
Addictive				0.461 (0.285-0.746);**0.002**

^a^ The chi-square test of goodness of fit was performed to check for Hardy–Weinberg equilibrium of the control group.

^b^ ORs, and 95% CIs, were calculated by unconditional logistic regression analysis, adjusted for age, Cigarette smoking, and Alcohol consumption.

The bold values indicate that the results are statistically significant.

### 3.5 Association of MYBL2 AS-related genetic variants with clinicopathological features of TNBC

We analysed the relationship of rs285170 and rs405660 with clinicopathological features of TNBC. No significant association between rs285170 and rs405660 was found with both tumour size (T) in TNBC (Table 5); however, rs405660 was associated with lymph node metastasis (N) under both dominant (OR = 0.396, 95% CI = 0.209–0.750, *p* = 0.005) and additive (OR = 0.390, 95% CI = 0.210-0.725, *p* = 0.003) models (Table 6). However, rs285170 was not associated with lymph node metastasis.

**TABLE 5:** Association of MYBL2 AS-related genetic variants with TNBC T staging.

Variable	Tumor size		OR (95%*CI*); *P* ^a^
	≤5 cm (N = 167) (%)	>5 cm (N = 28) (%)	
**rs285170**	167 (100.00)	28 (100.00)	
TT	147 (88.02)	24 (85.71)	1
TC	19 (11.38)	4 (14.29)	1.775 (0.528-5.973); 0.354
CC	1 (0.60)	0 (0.00)	--
Dominant			1.682 (0.503-5.631); 0.399
Addictive			1.512 (0.487-4.692); 0.474
**rs405660**	166 (99.40)	28 (100.00)	
GG	116 (69.88)	21 (75.00)	1
GT	48 (28.92)	7 (25.00)	0.914 (0.358-2.336); 0.851
TT	2 (1.20)	0 (0.00)	--
Dominant			0.889 (0.348-2.271); 0.806
Addictive			0.862 (0.347-2.139); 0.748

^a^ ORs, and 95% CIs, were calculated by unconditional logistic regression analysis.

**TABLE 6:** Association of MYBL2 AS-related genetic variants with TNBC N staging.

Variable	Node		OR (95%*CI*); *P* ^a^
	N0 (N = 87) (%)	≧N1(N = 108) (%)	
**rs285170**	87 (100.00)	108 (100.00)	
TT	76 (87.36)	95 (86.30)	1
TC	10 (11.49)	13 (13.70)	1.117 (0.458-2.725); 0.808
CC	1 (1.15)	0 (0.00)	--
Dominant			1.013 (0.424-2.422); 0.976
Addictive			0.921 (0.407-2.083); 0.842
**rs405660**	87 (100.00)	107 (99.07)	
GG	52 (59.77)	85 (79.44)	1
GT	33 (37.93)	22 (20.56)	0.417 (0.219-0.795);**0.008**
TT	2 (2.30)	0 (0.00)	--
Dominant			0.396 (0.209-0.750);**0.005**
Addictive			0.390 (0.210-0.725);**0.003**

^a^ ORs, and 95% CIs, were calculated by unconditional logistic regression analysis.

The bold values indicate that the results are statistically significant.

### 3.6 Analysis of biological function

**FIGURE 3:**
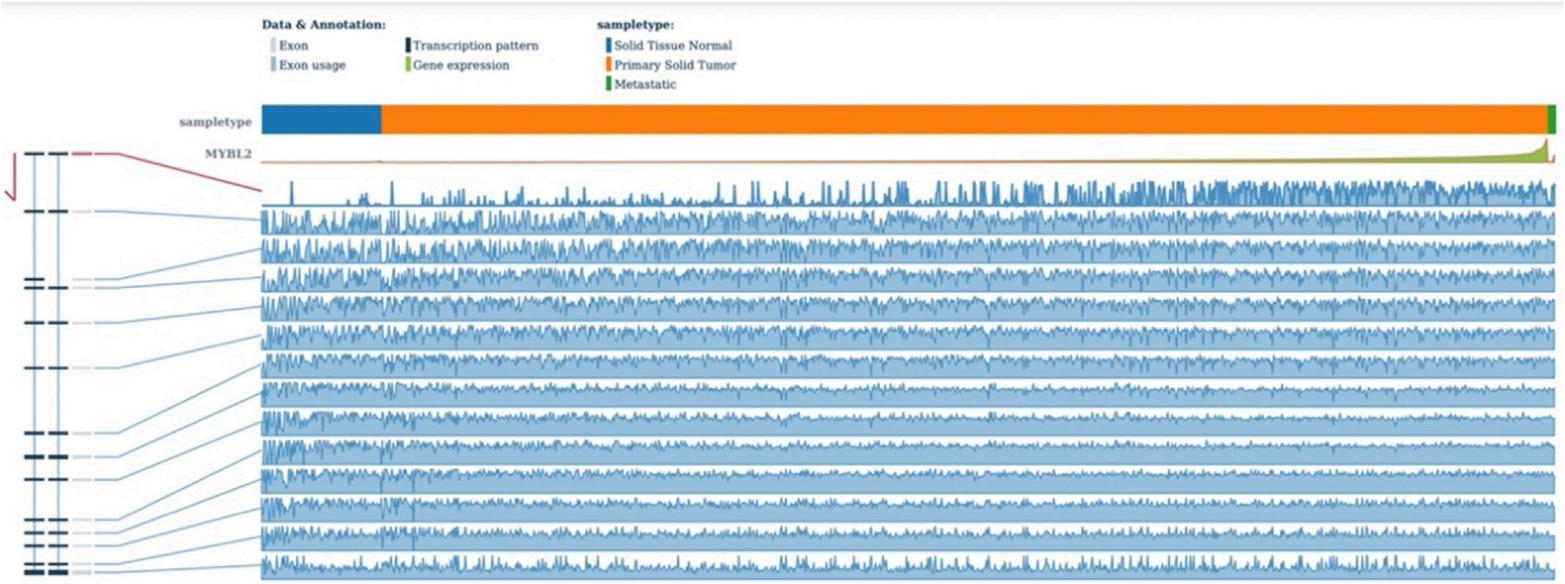
TSVdb outputs the expression of each exon of *MYBL2*.

**FIGURE 4:**
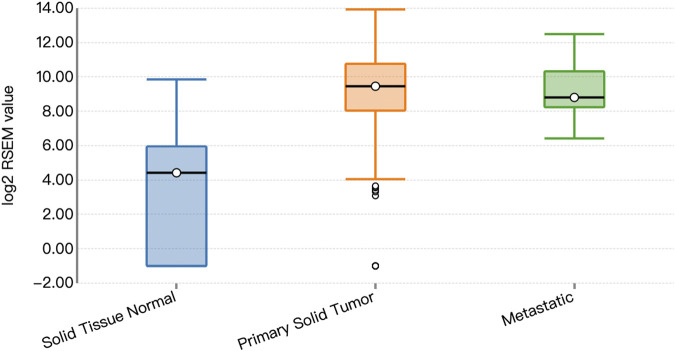
Whole exon spliceosome expression.

**FIGURE 5:**
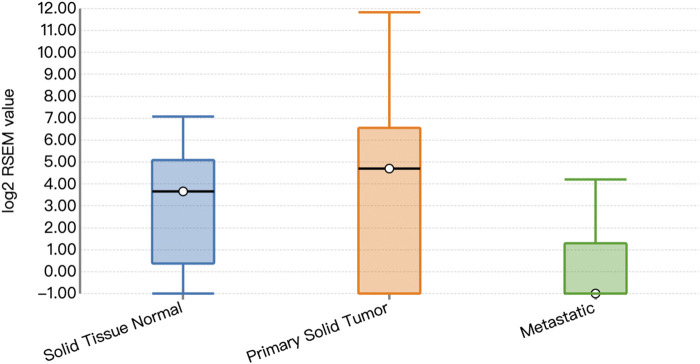
Expression of exon 3-skipped spliceosomes.

Functional prediction of our candidate SNPs in CancerSplicingQTL indicated that both rs285170 and rs405660 were associated with *MYBL2* AS. The splice site was chr20:42310424, and both affected *MYBL2* exon 3 skipping; that is, generation of a spliceosome with exon 3 skipping (Table 7). The expression landscape of *MYBL2* splice isoforms in breast cancer was next analysed on the TSVdb platform. In this database, we performed an analysis of the exonic expression landscape of *MYBL2* in breast cancer, with the final output shown in Figure 3. The figure illustrates the two splice isoforms of *MYBL2* and the 14 exons on *MYBL2* in normal, orthotopic, and metastatic breast cancer tissue, and the difference between the two splice isoforms is shown in Figure 3 as to whether exon 3 skipping occurs during the splicing process. The expression differences of the two splice isoforms between tissues are shown in Figures 4, 5. The expression of whole exon spliceosomes without AS was significantly higher in breast cancer than in normal tissues (Figure 4), while the expression of exon 3-skipped spliceosomes was not significantly different between breast cancer and normal tissues (Figure 5). This result suggests that the exon 3-skipped spliceosome does not increase breast cancer susceptibility and that this spliceosome reduces breast cancer susceptibility compared to whole exon spliceosome carriers.

**TABLE 7:** Splicing function prediction of candidate sites.

SNP position	SNP ID	Splicing type^a^	Splicing exon	Splicing position
20:42335680	rs285170	ES	3	20:42310424
20:42308715	rs405660	ES	3	20:42310424

^a^ ES, exon skip.

## 4 Discussion

TNBC is a highly proliferative subtype of breast cancer that is often associated with poor prognosis. The treatment strategy of breast cancer varies based on molecular typing; however, as TNBC lacks expression of ER, PR, and HER-2, it has low hormone sensitivity, and the prognostic markers and therapeutic targets remain unknown. Compared with other invasive breast cancers, TNBC has an earlier onset and a higher recurrence rate, and it is also more likely to spread and metastasise (Berger et al., 2021). TNBC is often difficult to treat owing to the lack of effective therapeutic agents, and to date, no targeted therapy has been approved by the US Food and Drug Administration (Zheng et al., 2020). Therefore, studies on TNBC susceptibility are particularly important. Only a few individuals with similar exposure to environmental risk factors for breast cancer eventually develop breast cancer, indicating that genetic factors play a key role. The genetic predisposition to breast cancer represents an important research focus in current studies. Classic *BRCA1*/*2* germline mutations are associated with approximately 15%–20% of TNBC cases; nevertheless, the susceptibility genes and mutation sites have only been specified in few patients with TNBC (Pohl-Rescigno et al., 2020).

*MYBL2* is an important regulatory gene in cell cycle progression and it affects cell proliferation and differentiation. Several studies have found an association of *MYBL2* with malignant tumour development, such as breast, bladder, lung adenocarcinoma, and ovarian cancer (Liu Q et al., 2022; Inoue and Fry, 2016; Lee et al., 2022; Liu W et al., 2022). The *MYBL2* S427G variant has been reported to be associated with an increased risk of basal-like breast cancer in the African American population; however, the specific mechanism has not been clarified (Thorner et al., 2009). Chen and Chen (2018) conducted a preliminary study on the mechanisms of *MYBL2* in breast cancer development and found that miR-143-3p targets the *MYBL2* 3′-untranslated region, which inhibits *MYBL2* expression and promotes proliferation and inhibits apoptosis of breast cancer cells. A *MYBL2* splice variant lacking exon 9A has been found to function as a suppressor protein and counteract transactivation mediated by the whole exon-expressed *MYBL2* (Horstmann et al., 2000). Although the study did not explore the effect of AS on cancer risk, several studies have shown that AS leads to an increase in cancer risk (Chan et al., 2022). Previous studies have explained part of the mechanism of MYBL2 in breast cancer risk; however, at present, no studies have systematically analysed the relationship between AS of *MYBL2* and breast cancer risk (Bayley et al., 2020). Therefore, we performed bioinformatics analysis and found that two functional SNPs, rs285170 and rs405660, are likely to cause AS in *MYBL2*. These two SNPs are likely to influence the risk of TNBC by affecting AS of *MYBL2*. To our knowledge, this is the first study conducted worldwide on these two SNPs and the first systematic analysis of the association of *MYBL2* AS-related genetic variants with AS risk.

We found that the C allele of rs285170 was associated with decreased susceptibility to TNBC in 217 of our cases, and the same results were confirmed in dominant and additive models. However, no significant difference was found in participants aged <50 years, and when this group of the cohort was excluded, the difference was more pronounced in the group aged ≧50 years. The G allele of rs405660 also exhibited a similar protective effect. A certain characteristic of the population aged ≧50 years is expected to play a role in this protective effect. Combined with the age characteristic analysis, we speculate that a joint effect relationship exists between menopausal status and mutations at this locus. After menopause, women experience certain changes in levels of hormones such as oestrogen and progesterone, and menopausal status is often found to be a risk factor for breast cancer (Howlader et al., 2014). ERβ expression is positively correlated with *MYBL2* expression in endometrial cancer tissues, which supports a relationship between the increased risk of endometrial cancer and *MYBL2* expression levels in premenopausal women (Luengo-Gil et al., 2019). Current studies have mostly focused on the relationship between hormone receptors and *MYBL2* expression; however, ER, PR, and HER-2 are not expressed in TNBC cases. Therefore, to explain whether *MYBL2* expression in TNBC is associated with hormone level changes, studies should confirm the existence of associations between hormone level changes and *MYBL2* expression. In addition, unlike rs285170, where the homozygous rs405660 variant showed significant protection in the full-age analysis, the association of the homozygous rs285170 variant with TNBC risk was not significant. This may be explained by the low MAF of rs285170 with few homozygous mutant genotypes in our sample. Follow-up studies using larger sample sizes may obtain significant results. In addition, we found that rs405660 was significantly associated with a decreased risk of lymph node metastasis in TNBC, and this finding may provide a reference for subsequent clinicopathological studies on MYBL2 in association with TNBC.

On the basis of population association analyses, we performed biological functional analyses based on TCGA data. The results showed that both rs285170 and rs405660 were associated with *MYBL2* exon 3 skipping. Whole exon spliceosomes significantly increase breast cancer susceptibility, but the exon 3-deleted spliceosome was not significantly different between breast cancer tissues and adjacent non-cancerous tissues. It reduced susceptibility to breast cancer compared with the whole exon spliceosome. This is consistent with the population association analysis results that *MYBL2* AS-related genetic variants can reduce the risk of breast cancer.

In this study, we proposed a novel bioinformatic analysis strategy to search for genetic variants associated with AS, systematically analysed all SNPs potentially causing AS in *MYBL2*, and determined linkage loci in the SNPs most likely to exhibit AS function. Our findings may provide a reference for AS-related research. Our study had a few limitations. First, our cases were all recruited as inpatients in hospitals, and there may be a certain Berkson’s bias. Secondly, since the AS-related SNPs, splicing exons, splicing sites, and splicing patterns obtained in this study were all predicted by bioinformatics, we recommend that subsequent studies supplement biological functional verification, such as analysis of SNP association with *MYBL2* splice isoform expression levels and minigene construct or subcutaneous tumour bearing experiments in nude mice.

To the best of our knowledge, this is the first study to systematically investigate the role of *MYBL2* AS-related genetic variants in TNBC susceptibility in the Chinese population using bioinformatics analysis. We found an association between rs285170 and rs405660 and the development of TNBC. The SNPs rs285170 and rs405660 may produce different splicing isoforms by altering the transcription of *MYBL2*, which in turn affects the risk of TNBC. In addition, we found that rs405660 was associated with the risk of lymph node metastasis in TNBC. Our findings elucidate the mechanisms underlying TNBC development and cancer in general.

## Data Availability

The original contributions presented in the study are included in the article/Supplementary Material, further inquiries can be directed to the corresponding authors.
