# Supplementary material for: MYBL2 alternative splicing-related genetic variants reduce the risk of triple-negative breast cancer in the Chinese population
Source: Front Genet. 2023 Apr 18;14:1150976. doi: 10.3389/fgene.2023.1150976 (PMC10151490; doi:10.3389/fgene.2023.1150976)
Supplement: Supplementary file 1 [file Table1.docx]

**Extended Table 1. CancerSplicingQTL database predicted SNPs affecting MYBL2 alternative splicing in breast cancer**

| SNP position | SNP ID | Alleles | HGVS cDNA |
| --- | --- | --- | --- |
| 20:42326880 | rs184032 | A/G | c.664-1517A>G |
| 20:42336684 | rs420755 | C/G | c.1506-1919C>G |
| 20:42340684 | rs285172 | G/A | c.1719+443G>A |
| 20:42336205 | rs826955 | C/G | c.1505+2207C>G |
| 20:42337875 | rs285171 | C/G | c.1506-728C>G |
| 20:42306971 | rs11470203 | GAAAAA/G | c.115-3452_115-3443del |
| 20:42307092 | rs826953 | A/T | c.115-3332A>T |
| 20:42306260 | rs826951 | A/G | c.114+3721A>G |
| 20:42306307 | rs3091245 | A/T | c.114+3768A>T |
| 20:42299914 | rs826947 | T/G | c.21-2532T>G |
| 20:42300725 | rs3127069 | T/C | c.21-1721T>C |
| 20:42312304 | rs376532 | G/C | c.279+778G>C |
| 20:42310509 | rs442143 | T/C | c.186+14T>C |
| 20:42311855 | rs387769 | A/G | c.279+329T>C |
| 20:42240397 | rs6093860 | C/A | c.486-2093C>A |
| 20:42312619 | rs3092364 | G/A | c.279+1093G>A |
| 20:42311088 | rs445912 | T/C | c.187-346T>C |
| 20:42238822 | rs6030981 | C/T | c.486-3668C>T |
| 20:42238857 | rs6030982 | G/A | c.486-3633G>A |
| 20:42239145 | rs2038457 | A/G | c.486-3345A>G |
| 20:42314067 | rs6031036 | T/C | c.280-1425T>C |
| 20:42329799 | rs285165 | A/G | c.951+1115C>T |
| 20:42294719 | rs826944 | T/C | -- |
| 20:42230695 | rs6073143 | C/T | c.208-1705T>C |
| 20:42306943 | rs140599625 | AAAAT/A | c.115-3480_115-3473del |
| 20:42227012 | rs6073142 | C/T | c.207+1850G>A |
| 20:42238250 | rs6103393 | A/T | c.486-4240A>T |
| 20:42237067 | rs2145214 | C/A | c.485+3354C>A |
| 20:42236697 | rs11427975 | A/AG | c.485+2984_485+2985insG |
| 20:42236671 | rs4812726 | A/G | c.485+2958A>G |
| 20:42243186 | rs6073152 | G/T | c.612+570G>T |
| 20:42236454 | rs4812725 | G/A | c.485+2741G>A |
| 20:42228984 | rs1986255 | A/G | c.208-3416A>G |
| 20:42316582 | rs285187 | G/A | c.500+870G>A |
| 20:42225923 | rs4810386 | A/G | c.207+761A>G |
| 20:42291961 | rs385345 | A/G | -- |
| 20:42253898 | rs6103396 | C/A | c.923+1213C>A |
| 20:42276644 | rs3091647 | A/G | -- |
| 20:42218214 | rs6065629 | T/C | -- |
| 20:42218919 | rs1894668 | C/G | -- |
| 20:42257994 | rs10557881 | GCTCC/G | c.923+5310_923+5313del |
| 20:42260247 | rs6073162 | T/C | c.924-4319T>C |
| 20:42221606 | rs6073139 | G/A | c.-6-1727G>A |
| 20:42262868 | rs6031003 | G/A | c.924-1698G>A |
| 20:42283990 | rs6103413 | G/C | -- |
| 20:42222395 | rs6093859 | G/T | c.-6-938G>T |
| 20:42242700 | rs4473450 | A/G | c.612+84C>T |
| 20:42264502 | rs6031007 | C/T | c.924-64G>A |
| 20:42286082 | rs397553 | G/A | -- |
| 20:42286264 | rs6017135 | C/T | -- |
| 20:42220368 | rs6017120 | A/G | c.-7+674A>G |
| 20:42281384 | rs285193 | A/G | n.183+39A>G |
| 20:42219221 | rs6065630 | T/C | -- |
| 20:42219265 | rs6073136 | T/C | -- |
| 20:42219780 | rs3752561 | G/T | c.-7+86G>T |
| 20:42255437 | rs138432349 | AAGTGTTGAT/A | c.923+2753_923+2761del |
| 20:42278139 | rs35481647 | ATTC/A | -- |
| 20:42281834 | rs285194 | A/G | n.338A>G |
| 20:42235829 | rs146734714 | T/C | c.485+2116T>C |
| 20:42244003 | rs6130427 | C/T | c.612+1387A>G |
| 20:42258960 | rs6030998 | C/T | c.924-5606C>T |
| 20:42282286 | rs285196 | T/A | -- |
| 20:42285359 | rs6093867 | G/T | -- |
| 20:42282471 | rs184033 | A/G | -- |
| 20:42285456 | rs6017134 | G/T | -- |
| 20:42286511 | rs6017136 | G/A | -- |
| 20:42286805 | rs2425618 | C/T | -- |
| 20:42288301 | rs2867730 | G/T | -- |
| 20:42279727 | rs826962 | C/T | -- |
| 20:42278268 | rs2867729 | G/A | -- |
| 20:42226487 | rs6065632 | C/T | c.207+1325C>T |
| 20:42279114 | rs285190 | G/A | -- |
| 20:42247156 | rs6073153 | C/T | c.613-424C>T |
| 20:42221633 | rs6073140 | T/G | c.-6-1700T>G |
| 20:42246284 | rs4810389 | A/G | c.613-1296A>G |
| 20:42265966 | rs6031008 | G/A | c.1120+73G>A |
| 20:42268030 | rs409587 | G/C | c.1120+2137G>C |
| 20:42244578 | rs11476681 | GA/G | c.612+1963del |
| 20:42308217 | rs454255 | T/G | c.115-2207T>G |
| 20:42308715 | rs405660 | T/G | c.115-1709T>G |
| 20:42309380 | rs428000 | A/G | c.115-1044A>G |
| 20:42227511 | rs4810387 | A/G | c.207+2349A>G |
| 20:42225114 | rs2664519 | G/A | c.159G>A |
| 20:42217422 | rs6030970 | G/T | -- |
| 20:42242943 | rs6073150 | C/T | c.612+327C>T |
| 20:42285890 | rs385609 | C/G | -- |
| 20:42285962 | rs412396 | C/G | -- |
| 20:42241347 | rs10646057 | A/ACAAT | c.486-1143_486-1142insCAAT |
| 20:42285948 | rs367342 | G/T | -- |
| 20:42219831 | rs3752562 | C/T | c.-7+137G>A |
| 20:42216647 | rs1055334 | A/G | c.*1166A>G |
| 20:42270921 | rs439115 | T/C | c.1121-198T>C |
| 20:42255864 | rs2093139 | G/A | c.923+3179G>A |
| 20:42256630 | rs4812728 | G/A | c.923+3945G>A |
| 20:42225677 | rs62225579 | A/G | c.207+515A>G |
| 20:42258140 | rs6030996 | C/A | c.923+5455C>A |
| 20:42251082 | rs11476747 | CA/C | c.769-1448_769-1447del |
| 20:42243805 | rs6065636 | T/C | c.612+1189T>C |
| 20:42243863 | rs6093861 | A/G | c.612+1247A>G |
| 20:42283166 | rs6073169 | G/C | -- |
| 20:42244259 | rs2903655 | C/T | c.612+1643C>T |
| 20:42244297 | rs1973954 | G/T | c.612+1681G>T |
| 20:42306309 | rs6130432 | T/C | c.114+3770T>C |
| 20:42296961 | rs285204 | T/C | c.20+1018T>C |
| 20:42297411 | rs285201 | C/T | c.20+1468C>T |
| 20:42300262 | rs826948 | A/G | c.21-2184A>G |
| 20:42315091 | rs285186 | G/A | c.280-401G>A |
| 20:42252325 | rs4810390 | G/T | c.769-206G>T |
| 20:42248666 | rs6030988 | C/T | c.700-831C>T |
| 20:42253542 | rs6073157 | A/G | c.923+857A>G |
| 20:42217085 | rs6103390 | T/C | -- |
| 20:42263113 | rs6065637 | T/G | c.924-1453T>G |
| 20:42263872 | rs6130429 | A/G | c.924-694A>G |
| 20:42264726 | rs2273523 | T/C | c.1011+73T>C |
| 20:42217965 | rs375176196 | C/CTTTTTTTT | -- |
| 20:42263810 | rs6031004 | A/C | c.924-756A>C |
| 20:42263927 | rs6031005 | A/G | c.924-639A>G |
| 20:42264017 | rs6031006 | G/A | c.924-549G>A |
| 20:42216887 | rs6030969 | A/G | -- |
| 20:42243121 | rs6073151 | G/A | c.612+505G>A |
| 20:42246198 | rs4810388 | C/G | c.613-1382C>G |
| 20:42265650 | rs6017129 | A/G | c.1012-135A>G |
| 20:42268608 | rs2867728 | C/T | c.1121-2511C>T |
| 20:42269744 | rs427205 | A/G | c.1121-1375A>G |
| 20:42270837 | rs383634 | C/T | c.1121-282C>T |
| 20:42271374 | rs426197 | T/G | c.1266+110T>G |
| 20:42247288 | rs370439895 | A/ATT | -- |
| 20:42268341 | rs422234 | C/T | c.1120+2448C>T |
| 20:42226506 | rs6103391 | G/C | c.207+1344G>C |
| 20:42249185 | rs6073155 | C/A | c.700-312C>A |
| 20:42248848 | rs6073154 | T/A | c.700-649T>A |
| 20:42234936 | rs6130425 | T/C | c.485+1223T>C |
| 20:42230644 | rs6030978 | C/T | c.208-1756C>T |
| 20:42214525 | rs6073134 | C/A | c.*803+316C>A |
| 20:42271804 | rs142320243 | C/CA | c.1266+541del |
| 20:42225682 | rs62225580 | G/C | c.207+520G>C |
| 20:42215474 | rs6093858 | G/A | c.*804-811G>A |
| 20:42222984 | rs6030975 | C/T | c.-6-349T>C |
| 20:42240730 | rs6103395 | C/T | c.486-1760G>A |
| 20:42289962 | rs826940 | T/C | -- |
| 20:42277392 | rs3092649 | C/A | -- |
| 20:42221300 | rs6030974 | G/A | c.-7+1606G>A |
| 20:42290148 | rs826941 | C/T | -- |
| 20:42281914 | rs285195 | A/G | n.418A>G |
| 20:42285353 | rs6093866 | T/C | -- |
| 20:42238931 | rs11324612 | CA/C | c.486-3558_486-3551del |
| 20:42238952 | rs34148938 | TA/T | c.486-3537del |
| 20:42282116 | rs3091584 | A/AAGTT | -- |
| 20:42250442 | rs6030990 | C/T | c.768+877C>T |
| 20:42331846 | rs7347231 | C/T | c.1365+303C>T |
| 20:42259636 | rs714998 | T/C | c.924-4930T>C |
| 20:42265700 | rs6065638 | T/C | c.1012-85T>C |
| 20:42269858 | rs439560 | T/G | c.1121-1261T>G |
| 20:42249398 | rs1555348 | C/T | c.700-99C>T |
| 20:42253855 | rs6030993 | A/T | c.923+1170A>T |
| 20:42248622 | rs6030987 | C/T | c.700-875C>T |
| 20:42357295 | rs3127065 | A/C | -- |
| 20:42335680 | rs285170 | C/T | c.1505+1682C>T |
| 20:42324218 | rs3091248 | A/G | c.663+3259A>G |
| 20:42317419 | rs285188 | C/T | c.500+1707A>G |
| 20:42329945 | rs285166 | G/A | c.952-1185G>A |
| 20:42330576 | rs285168 | T/C | c.952-554T>C |
| 20:42326577 | rs3117538 | G/A | c.664-1820G>A |
| 20:42321222 | rs3117537 | A/G | c.663+263A>G |
| 20:42321828 | rs384132 | C/A | c.663+869C>A |
| 20:42323034 | rs3092442 | A/C | c.663+2075A>C |
| 20:42323036 | rs3092195 | T/TTA | c.663+2077_663+2078insTA |
| 20:42323054 | rs3091247 | C/A | c.663+2095C>A |
| 20:42345960 | rs285176 | C/T | -- |
| 20:42349848 | rs285184 | G/A | -- |
| 20:42346207 | rs285177 | T/G | -- |
| 20:42328749 | rs285163 | A/G | c.951+65A>G |
| 20:42328222 | rs285161 | C/G | c.664-175G>C |
| 20:42342721 | rs285173 | C/T | c.1824+975C>T |
| 20:42313414 | rs547151 | T/C | c.279+1888T>C |
| 20:42313441 | rs608805 | T/C | c.279+1915T>C |
| 20:42313552 | rs3092729 | T/C | c.280-1940T>C |
| 20:42313478 | rs3091560 | TA/T | c.279+1953_279+1954del |
| 20:42345463 | rs285175 | T/C | -- |
| 20:42328639 | rs285162 | T/C | c.906T>C |
| 20:42329964 | rs285167 | A/G | c.952-1166A>G |
| 20:42332782 | rs285169 | C/T | c.1366-1077C>T |
| 20:42309154 | rs436397 | T/C | c.115-1270T>C |
| 20:42343027 | rs166941 | C/G | c.1825-747C>G |
| 20:42307712 | rs826954 | A/G | c.115-2712A>G |
| 20:42333293 | rs6017147 | C/G | c.1366-566C>G |
| 20:42300481 | rs3117536 | T/G | c.21-1965T>G |
| 20:42319516 | rs166942 | G/A | c.501-1281G>A |
| 20:42311222 | rs439730 | A/C | c.187-212A>C |
| 20:42317440 | rs285189 | T/C | c.500+1728T>C |
| 20:42319624 | rs188219 | G/T | c.501-1173C>A |
| 20:42237502 | rs6124583 | T/C | c.485+3789T>C |
| 20:42239100 | rs6103394 | G/A | c.486-3390G>A |
| 20:42218309 | rs11439550 | C/CT | -- |
| 20:42290257 | rs826942 | T/A | -- |
| 20:42277818 | rs6031017 | T/C | -- |
| 20:42279142 | rs285191 | A/G | -- |
| 20:42275499 | rs393115 | G/T | c.1267-77G>T |
| 20:42256865 | rs4812729 | A/G | c.923+4180A>G |
