# Supplementary material for: MYBL2 alternative splicing-related genetic variants reduce the risk of triple-negative breast cancer in the Chinese population
Source: Front Genet. 2023 Apr 18;14:1150976. doi: 10.3389/fgene.2023.1150976 (PMC10151490; doi:10.3389/fgene.2023.1150976)
Supplement: Supplementary file 4 [file Table2.docx]

**Extended Table 2.** Haploview linkage disequilibrium analysis results.

| Allele | Test | r^2^ |
| --- | --- | --- |
| 20:42214525 | 20:42222984 | 0.884 |
| 20:42215474 | 20:42222984 | 0.971 |
| 20:42216647 | 20:42222984 | 0.971 |
| 20:42216887 | 20:42222984 | 0.971 |
| 20:42217085 | 20:42222984 | 0.971 |
| 20:42217422 | 20:42222984 | 0.971 |
| 20:42218214 | 20:42222984 | 0.971 |
| 20:42218919 | 20:42222984 | 0.971 |
| 20:42219221 | 20:42222984 | 0.971 |
| 20:42219265 | 20:42222984 | 0.971 |
| 20:42219780 | 20:42222984 | 1 |
| 20:42219831 | 20:42222984 | 1 |
| 20:42220368 | 20:42222984 | 1 |
| 20:42221300 | 20:42222984 | 1 |
| 20:42221606 | 20:42222984 | 1 |
| 20:42221633 | 20:42222984 | 1 |
| 20:42222395 | 20:42222984 | 1 |
| 20:42222984 | 20:42222984 | 1 |
| 20:42225114 | 20:42222984 | 1 |
| 20:42225677 | 20:42222984 | 1 |
| 20:42225682 | 20:42222984 | 1 |
| 20:42225923 | 20:42222984 | 1 |
| 20:42226487 | 20:42222984 | 1 |
| 20:42226506 | 20:42222984 | 1 |
| 20:42227012 | 20:42222984 | 1 |
| 20:42227511 | 20:42222984 | 1 |
| 20:42228984 | 20:42222984 | 1 |
| 20:42230644 | 20:42222984 | 1 |
| 20:42230695 | 20:42222984 | 1 |
| 20:42234936 | 20:42222984 | 1 |
| 20:42235829 | 20:42222984 | 1 |
| 20:42236454 | 20:42222984 | 1 |
| 20:42236671 | 20:42222984 | 1 |
| 20:42237067 | 20:42222984 | 1 |
| 20:42237502 | 20:42256865 | 0.955 |
| 20:42238250 | 20:42222984 | 1 |
| 20:42238822 | 20:42222984 | 1 |
| 20:42238857 | 20:42222984 | 1 |
| 20:42239100 | 20:42256865 | 1 |
| 20:42239145 | 20:42222984 | 1 |
| 20:42240397 | 20:42222984 | 1 |
| 20:42240730 | 20:42240730 | 1 |
| 20:42242700 | 20:42222984 | 1 |
| 20:42242943 | 20:42222984 | 1 |
| 20:42243121 | 20:42222984 | 1 |
| 20:42243186 | 20:42222984 | 1 |
| 20:42243805 | 20:42222984 | 1 |
| 20:42243863 | 20:42222984 | 1 |
| 20:42244003 | 20:42222984 | 1 |
| 20:42244259 | 20:42222984 | 1 |
| 20:42244297 | 20:42222984 | 1 |
| 20:42246198 | 20:42222984 | 1 |
| 20:42246284 | 20:42222984 | 1 |
| 20:42247156 | 20:42222984 | 1 |
| 20:42248622 | 20:42222984 | 1 |
| 20:42248666 | 20:42222984 | 1 |
| 20:42248848 | 20:42222984 | 1 |
| 20:42249185 | 20:42222984 | 1 |
| 20:42249398 | 20:42222984 | 1 |
| 20:42250442 | 20:42222984 | 1 |
| 20:42252325 | 20:42222984 | 1 |
| 20:42253542 | 20:42222984 | 0.942 |
| 20:42253855 | 20:42222984 | 1 |
| 20:42253898 | 20:42222984 | 1 |
| 20:42255864 | 20:42222984 | 1 |
| 20:42256630 | 20:42222984 | 1 |
| 20:42256865 | 20:42256865 | 1 |
| 20:42258140 | 20:42222984 | 1 |
| 20:42258960 | 20:42222984 | 1 |
| 20:42259636 | 20:42222984 | 0.971 |
| 20:42260247 | 20:42222984 | 0.971 |
| 20:42262868 | 20:42222984 | 0.971 |
| 20:42263113 | 20:42222984 | 1 |
| 20:42263810 | 20:42222984 | 0.971 |
| 20:42263872 | 20:42222984 | 1 |
| 20:42263927 | 20:42222984 | 0.971 |
| 20:42264017 | 20:42222984 | 1 |
| 20:42264502 | 20:42222984 | 0.971 |
| 20:42264726 | 20:42222984 | 0.971 |
| 20:42265650 | 20:42222984 | 0.971 |
| 20:42265700 | 20:42222984 | 0.971 |
| 20:42265966 | 20:42222984 | 1 |
| 20:42268030 | 20:42222984 | 0.971 |
| 20:42268341 | 20:42222984 | 0.971 |
| 20:42268608 | 20:42222984 | 0.971 |
| 20:42269744 | 20:42222984 | 0.971 |
| 20:42269858 | 20:42222984 | 0.971 |
| 20:42270837 | 20:42222984 | 0.971 |
| 20:42270921 | 20:42222984 | 0.971 |
| 20:42271374 | 20:42222984 | 0.971 |
| 20:42275499 | 20:42256865 | 1 |
| 20:42276644 | 20:42222984 | 0.971 |
| 20:42277392 | 20:42222984 | 0.971 |
| 20:42277818 | 20:42256865 | 1 |
| 20:42278268 | 20:42222984 | 0.971 |
| 20:42279114 | 20:42222984 | 0.971 |
| 20:42279142 | 20:42256865 | 1 |
| 20:42279727 | 20:42222984 | 0.971 |
| 20:42281384 | 20:42222984 | 0.971 |
| 20:42281834 | 20:42222984 | 0.971 |
| 20:42281914 | 20:42222984 | 0.971 |
| 20:42282286 | 20:42222984 | 0.971 |
| 20:42282471 | 20:42222984 | 0.971 |
| 20:42283166 | 20:42222984 | 0.971 |
| 20:42283990 | 20:42222984 | 0.971 |
| 20:42285353 | 20:42222984 | 0.971 |
| 20:42285359 | 20:42222984 | 0.971 |
| 20:42285456 | 20:42222984 | 0.971 |
| 20:42285890 | 20:42222984 | 0.971 |
| 20:42285948 | 20:42222984 | 0.971 |
| 20:42285962 | 20:42222984 | 0.971 |
| 20:42286082 | 20:42222984 | 0.971 |
| 20:42286264 | 20:42222984 | 0.971 |
| 20:42286511 | 20:42222984 | 0.971 |
| 20:42286805 | 20:42222984 | 0.971 |
| 20:42288301 | 20:42222984 | 0.971 |
| 20:42289962 | 20:42222984 | 0.971 |
| 20:42290148 | 20:42222984 | 0.971 |
| 20:42290257 | 20:42256865 | 1 |
| 20:42291961 | 20:42222984 | 0.942 |
| 20:42294719 | 20:42222984 | 0.942 |
| 20:42296961 | 20:42222984 | 0.942 |
| 20:42297411 | 20:42222984 | 0.942 |
| 20:42299914 | 20:42222984 | 0.942 |
| 20:42300262 | 20:42222984 | 0.942 |
| 20:42300481 | 20:42256865 | 0.953 |
| 20:42300725 | 20:42222984 | 0.942 |
| 20:42306260 | 20:42222984 | 0.942 |
| 20:42306307 | 20:42222984 | 0.942 |
| 20:42306309 | 20:42222984 | 0.942 |
| 20:42307092 | 20:42222984 | 0.942 |
| 20:42307712 | 20:42256865 | 0.953 |
| 20:42308217 | 20:42222984 | 0.942 |
| 20:42308715 | 20:42222984 | 0.942 |
| 20:42309154 | 20:42256865 | 0.953 |
| 20:42309380 | 20:42222984 | 0.942 |
| 20:42310509 | 20:42222984 | 0.942 |
| 20:42311088 | 20:42222984 | 0.942 |
| 20:42311222 | 20:42256865 | 0.953 |
| 20:42311855 | 20:42222984 | 0.942 |
| 20:42312304 | 20:42222984 | 0.942 |
| 20:42312619 | 20:42222984 | 0.942 |
| 20:42313414 | 20:42256865 | 0.953 |
| 20:42313441 | 20:42256865 | 0.953 |
| 20:42313552 | 20:42256865 | 0.953 |
| 20:42314067 | 20:42222984 | 0.942 |
| 20:42315091 | 20:42222984 | 0.942 |
| 20:42316582 | 20:42222984 | 0.942 |
| 20:42317419 | 20:42256865 | 0.953 |
| 20:42317440 | 20:42256865 | 0.953 |
| 20:42319516 | 20:42256865 | 0.953 |
| 20:42319624 | 20:42256865 | 0.953 |
| 20:42321222 | 20:42256865 | 0.953 |
| 20:42321828 | 20:42256865 | 0.953 |
| 20:42323034 | 20:42256865 | 0.953 |
| 20:42323054 | 20:42256865 | 0.953 |
| 20:42324218 | 20:42256865 | 0.953 |
| 20:42326577 | 20:42256865 | 0.953 |
| 20:42326880 | 20:42222984 | 0.942 |
| 20:42328222 | 20:42256865 | 0.953 |
| 20:42328639 | 20:42256865 | 0.953 |
| 20:42328749 | 20:42256865 | 0.953 |
| 20:42329799 | 20:42222984 | 0.942 |
| 20:42329945 | 20:42256865 | 0.953 |
| 20:42329964 | 20:42256865 | 0.953 |
| 20:42330576 | 20:42256865 | 0.953 |
| 20:42331846 | 20:42331846 | 1 |
| 20:42332782 | 20:42256865 | 0.953 |
| 20:42333293 | 20:42333293 | 1 |
| 20:42335680 | 20:42256865 | 0.907 |
| 20:42336205 | 20:42222984 | 0.942 |
| 20:42336684 | 20:42222984 | 0.942 |
| 20:42337875 | 20:42222984 | 0.942 |
| 20:42340684 | 20:42222984 | 0.942 |
| 20:42342721 | 20:42256865 | 0.907 |
| 20:42343027 | 20:42256865 | 0.907 |
| 20:42345463 | 20:42256865 | 0.907 |
| 20:42345960 | 20:42256865 | 0.907 |
| 20:42346207 | 20:42256865 | 0.907 |
| 20:42349848 | 20:42256865 | 0.907 |
| 20:42357295 | 20:42357295 | 1 |
