# Supplementary material for: MYBL2 alternative splicing-related genetic variants reduce the risk of triple-negative breast cancer in the Chinese population
Source: Front Genet. 2023 Apr 18;14:1150976. doi: 10.3389/fgene.2023.1150976 (PMC10151490; doi:10.3389/fgene.2023.1150976)
Supplement: Supplementary file 5 [file Table3.docx]

**Extended Table 3.** HSF functional prediction results

| SNP ID | Ref | Alt | Gene | Effect | HSF score |
| --- | --- | --- | --- | --- | --- |
| rs6073151 | A | G | IFT52 | ESE Site Broken |  |
| rs6103391 | C | G | IFT52 | ESS Site Broken |  |
| rs146734714 | C | T | IFT52 | ESS Site Broken |  |
| rs6103396 | A | C | IFT52 | ESS Site Broken |  |
| rs439560 | G | T | IFT52 | ESS Site Broken |  |
| rs2664519 | A | G | IFT52 | New ESE Site |  |
| rs6130425 | C | T | IFT52 | New ESE Site |  |
| rs6030982 | A | G | IFT52 | New ESE Site |  |
| rs6065636 | C | T | IFT52 | New ESE Site |  |
| rs714998 | C | T | IFT52 | New ESE Site |  |
| rs426197 | G | T | IFT52 | New ESE Site |  |
| rs6073143 | C | T | IFT52 | New ESS Site |  |
| rs409587 | C | G | IFT52 | New ESS Site |  |
| rs3092364 | A | G | MYBL2 | EIE (New ESE Site)/HSF Acceptor site | 41.09 > 68.96 => 67.83% |
| rs3117536 | G | T | MYBL2 | ESE Site Broken |  |
| rs454255 | G | T | MYBL2 | ESE Site Broken |  |
| rs285186 | A | G | MYBL2 | ESE Site Broken |  |
| rs3092442 | C | A | MYBL2 | ESE Site Broken |  |
| rs3127069 | C | T | MYBL2 | ESS Site Broken |  |
| rs445912 | C | T | MYBL2 | ESS Site Broken |  |
| rs387769 | C | T | MYBL2 | ESS Site Broken |  |
| rs3092729 | C | T | MYBL2 | ESS Site Broken |  |
| rs285189 | C | T | MYBL2 | ESS Site Broken |  |
| rs285162 | C | T | MYBL2 | ESS Site Broken |  |
| rs405660 | G | T | MYBL2 | HSF Acceptor site | 57.82 > 85.69 => 48.2% |
| rs384132 | A | C | MYBL2 | HSF Acceptor site | 41.65 > 69.52 => 66.91% |
| rs285171 | G | C | MYBL2 | HSF Acceptor site | 40.13 > 68 => 69.45% |
| rs285166 | A | G | MYBL2 | HSF Acceptor site/HSF Donor site | 44.02 > 71.89 => 63.31%/69.76 > 80.06 => 14.76% |
| rs285167 | G | A | MYBL2 | HSF Acceptor site/HSF Donor site | 47.9 > 75.77 => 58.18%/39.19 > 66.33 => 69.25% |
| rs285169 | T | C | MYBL2 | HSF Donor site | 37.96 > 65.1 => 71.5% |
| rs285170 | T | C | MYBL2 | HSF Donor site | 52.62 > 79.76 => 51.58% |
| rs826955 | G | C | MYBL2 | HSF Donor site | 38.18 > 65.32 => 71.08% |
| rs420755 | G | C | MYBL2 | HSF Donor site | 51.61 > 78.75 => 52.59% |
| rs285168 | C | T | MYBL2 | MaxEnt Donor site | 1.29 > 3.34 => 158.91% |
| rs285204 | C | T | MYBL2 | New ESE Site |  |
| rs6130432 | C | T | MYBL2 | New ESE Site |  |
| rs436397 | C | T | MYBL2 | New ESE Site |  |
| rs285187 | A | G | MYBL2 | New ESE Site |  |
| rs166942 | A | G | MYBL2 | New ESE Site |  |
| rs285161 | C | G | MYBL2 | New ESE Site |  |
| rs547151 | C | T | MYBL2 | New ESS Site |  |
